# Characterization of transcription factor MYB59 and expression profiling in response to low K^+^ and NO_3_^−^ in indica rice (*Oryza sativa* L.)

**DOI:** 10.1186/s43141-021-00248-6

**Published:** 2021-10-26

**Authors:** Md. Qamrul Islam, Md. Nazmul Hasan, Hammadul Hoque, Nurnabi Azad Jewel, Md. Fahmid Hossain Bhuiyan, Shamsul H. Prodhan

**Affiliations:** Department of Genetic Engineering and Biotechnology, Shahjalal University of Science and Technology, Sylhet, 3114 Bangladesh

**Keywords:** Potassium, Nitrogen, Transcription factor, Phylogeny, OsMYB59, 3D structure, RT-qPCR

## Abstract

**Background:**

Nitrogen and potassium are crucial supplements for plant development and growth. Plants can detect potassium and nitrate ions in soils and in like way, they modify root-to-shoot transport of these ions to adjust the conveyance among roots and shoots. Transcription factor MYB59 plays essential roles in numerous physiological processes inclusive of hormone response, abiotic stress tolerance, plant development, and metabolic regulation. In this study, we retrieved 56 MYB59 proteins from different plant species. Multiple sequence alignment, phylogenetic tree, conserved motif, chromosomal localization, and cis-regulatory elements of the retrieved sequences were analyzed. Gene structure, protein 3D structure, and DNA binding of OsMYB59 indica were also predicted. Finally, we characterized OsMYB59 and its function under low K^+^/NO_3_^−^ conditions in *Oryza sativa* subsp. *indica*.

**Results:**

Data analysis showed that MYB59s from various groups separated in terms of conserved functional domains and gene structure, where members of genus *Oryza* clustered together. Plants showed reduced height and yellowish appearance when grown on K^+^ and NO_3_^−^ deficient medium. Quantitative real-time PCR uncovered that the *OsMYB59* reacted to abiotic stresses where its expression was increased in BRRI dhan56 but decreased in other varieties on K^+^ deficient medium. In addition, *OsMYB59* transcript level increased on NO_3_^−^ deficient medium.

**Conclusions:**

Our results can help to explain the biological functions of indica rice MYB59 protein and gave a theoretical premise to additionally describe its biological roles in response to abiotic stresses particularly drought.

**Supplementary Information:**

The online version contains supplementary material available at 10.1186/s43141-021-00248-6.

## Background

Abiotic stress, such as drought, submergence, salt, temperature, and other environmental extremes, affects the majority of plants. Plants establish unique ways to adapt to severe climatic conditions in order to compensate for these circumstances. Membrane transport and perception systems play crucial and necessary roles in maintaining cell homeostasis under stressful situations [39]. Drought stress limits the development of roots in plants and the diffusion rates of potassium ions (K^+^) toward the roots in the soil, restricting the K uptake. During this reduction in K^+^ levels, plants resilience to drought stress and K^+^ absorption might be further depressed. Consequently, it is essential to maintain enough plant K for drought tolerance [54]. Also, overexpressing the potassium channels (such as AKT1) in rice has impacts on plants’ tolerance to drought [2]. On the other hand, nitrate (NO_3_^−^) is the most common type of nitrogen that plants utilize for development and growth. Plants obtain nitrogen by either atmospheric nitrogen fixation or by using N sources found in soil, such as nitrate, ammonium, urea, and organic forms of N. Nitrogen availability may influence drought tolerance in different ways. Under optimal water conditions and a high nitrate supply, there is enhanced root water absorption. Again, nitrate is associated with stomatal conductance and aquaporin expression, which may regulate plant adaptation to low water supply ([62]; Ding et al. 2016 [8]; Ding et al. 2018 [9]).

Potassium and nitrate ions enter into the plant by different transporters and channels, which convey K^+^ and NO_3_^−^ directly into root cells. In *Arabidopsis thaliana*, K^+^ transporters such as High Affinity K^+^ Transporter5 (HAK5) and K^+^ Absorption Permease7 (KUP7), as well as the K^+^ channel—*Arabidopsis* K^+^ Transporter1 (KT1), are important proteins involved in K^+^ uptake into the plant cells [17, 45, 48]. On the other hand, the assimilation of NO_3_^−^ is intervened by the Nitrate Transporter 1/Peptide Transporter (NRT1/PTR) and the transporters of Nitrate Transporter 2 (NRT2) families [27, 55, 63]. After absorption into root cells, K^+^ and NO_3_^−^ are released into xylem vessels and therefore transported toward the shoot. Root to shoot K^+^ transportation, in *Arabidopsis*, is mediated by the K^+^ transporter KUP7 and the K^+^ channel—Stelar K^+^ Outward Rectifier (SKOR). The Nitrate Transporter 1.5 (NRT1.5) is engaged with root to shoot NO_3_^−^ transport in *Arabidopsis* [32], and is currently called as Nitrate Transporter/Peptide Transporter Family 7.3 (NPF7.3) from the NRT1/PTR family. As a proton coupled K^+^ antiporter, NPF7.3 can also prevent K^+^ translocation from root to shoot [29]. Thus, in *Arabidopsis*, NPF7.3 governs the coordinated transport of K^+^ and NO_3_^−^ from the root to the shoot [10, 36]. Meanwhile, regulation of *SKOR* and *NPF7.3* mRNA are done at the level of transcription and the inclusion of NO_3_^−^ upregulates both *SKOR* and *NPF7.3* transcripts. When K^+^ levels are low, the transcript level of *NPF7.3* is reduced, preventing K^+^/NO_3_^−^ transport from root to shoot [29]. Thus, transcriptional control of both *SKOR* and *NPF7.3* might accomplish the coordinated transport of K^+^/NO_3_^−^ from root to shoot in *Arabidopsis.* In rice (*Oryza sativa*), there are several key transporters involved into K^+^ and NO_3_^−^ translocation such as OsHAK, OsKAT, OsHKT (for K^+^ transport) and OsNPF2.4, OsNPF2.2, OsNAR2.1, OsNRT2.3a (for NO_3_^−^ transport).

Transcription factors regulate gene expression and thereby govern a variety of essential biological processes. They can be classified into different families based on the characteristics of their DNA binding domains [47]. Transcription factors that contain DNA binding MYB domain comprise a large family that have a variety of roles in eukaryotes [15]. MYB type transcription factors share similar domain architecture. At the N-terminus, they have a DNA-binding MYB domain comprising 1 to 4 imperfect repeats residues and at the C-terminus, they have transcription activation or repression domain. Repeats of the MYB domain have a structure of helix-turn-helix (HTH) and each repeat is about 52 amino acids long. Moreover, MYB transcription factors are divided into four types: 1R, R2R3, 3R, and 4R, each with one to four repeats [12]. Although R2R3 MYB proteins are only found in terrestrial plants, they make up the largest subfamily of MYB type transcription factors [15]. In *Arabidopsis thaliana*, for example, more than 100 R2R3 MYB proteins have been identified. The R2R3-MYB proteins include important roles in cell metabolism, determination of cell fate, growth, and biotic/abiotic stress responses. Many R2R3-MYB proteins, like *Arabidopsis* AtMYB32 [44], *Triticum aestivum* TaMYB1D [57], *Prunus persica* PpMYB18 [67], *Chrysanthemum morifolium* CmMYB1 [50], and *Pinus taeda* PtMYB14 [4], are used for flavonoid and lignin synthesis in different plant species. *Oryza sativa* has 230 MYB proteins where *Arabidopsis* has 339 MYB proteins [15]. They perform major functions such as hormone response, abiotic stress tolerance, plant growth, and metabolic control in various physiological processes [12]. In *Arabidopsis*, the MYB59 DNA binding domain interacts with the promoter of *NPF7.3*, resulting in increased *NPF7.3* expression in response to outer environment K^+^/NO_3_^−^ levels. *NPF7.3* and *MYB59* function along the same pathway for directing root to shoot K^+^/NO_3_^−^ transport.

To manage stress conditions, many molecular changes can take place by activating and regulating certain stress-related genes [39]. Identifying important and differentially expressed genes in response to stress environments might be a suitable approach toward a better understanding of stress responses and their mechanisms [23]. Past studies indicated that rice *MYB59* is positively regulated with drought [46] and there are little studies about the regulation of *OsMYB* transcripts under K^+^ and NO_3_^−^ stress. Transcription factor OsMYB59 indica is significantly less studied and its function is still to be validated. Under water deficient conditions, *OsMYB59* may be upregulated and as previously described, K^+^/NO_3_^−^ are closely associated with drought. Recently, functional validation and expression of MYB59 in *Arabidopsis* under K^+^ and NO_3_^−^ deficient conditions were studied [11]. So, characterization and expression profiling of OsMYB59 indica were carried out in this study to know more about the role of this transcription factor.

## Methods

### Sequence retrieval of transcription factor MYB59 in different plant species, conserved motif analysis, multiple sequence alignment, and phylogenetic analysis

The amino acid, cDNA, CDS sequences, and corresponding annotations were downloaded from the NCBI (http://www.ncbi.nih.nim.gov) and EnsemblPlants (http://plants.ensembl.org/index.html) (Supplementary Table S1). Sequences from 56 plant species were retrieved and saved in a text file.

Conserved motifs were identified using the Meme program (http://meme-suite.org/index.html) [3] with statistical significance. The Meme program was run with default settings except that the maximum number of motifs was defined as 3 and the maximum width was set to 50.

Protein sequences of MYB59 identified from 56 plant species were aligned using Clustal Omega [52], with default parameters. The identical and similar residues of the alignment were shaded using the BoxShade Server (https://embnet.vital-it.ch/software/BOX_form.html) by ExPASy.

A multiple alignment was performed with the full-length amino acid sequences of MYB59 proteins using MEGA-X v. 10.1.7 (https://www.megasoftware.net/) [25]. Unrooted trees were constructed by the maximum-likelihood (ML) method with the following parameters: Poisson model; partial deletion; 1000 bootstrap replicates.

### Chromosomal location, gene structure, and cis-element analysis of rice *MYB59*

All required data regarding chromosomal location of rice *MYB59* was retrieved from EnsemblPlants (Accession no: BGIOSGA005283-TA > Location > Region in detail) and mapping was prepared using Microsoft Powerpoint (Office 365), while the gene structure was determined using Gene Structure Display Server 2.0 (http://gsds.cbi.pku.edu.cn/) [21]. We used PlantCARE database (http://bioinformatics.psb.ugent.be/webtools/plantcare /html/) [28] to find out cis-elements.

### Analysis of protein features

Physico-chemical features of OsMYB59 were analyzed by ProtParam tool (http://web. expasy.org/protparam/) [16] and sub-cellular localization was predicted by CELLO server (http://cello.life.nctu.edu.tw/) [64].

3D models were generated by SWISS-MODEL (https://swissmodel.expasy.org/) [49], refined by ModRefiner (https://zhanglab.ccmb.med.umich.edu/ModRefiner/) [58], validated by Ramachandran plot (https://servicesn.mbi.ucla.edu/PROCHECK/), and visualized by PyMol (https://pymol.org/2/) [7]. Template 6kks.1.A [53] was used because it has an identity of 52.78% with our target sequence. MYB59 protein and DNA (B-DNA) docking was done using HDOCK SERVER (http://hdock.phys.hust.edu.cn/) [60].

### Plant materials, growth conditions, and treatments

In this experiment, a total of three indica rice genotypes were used. Mature seeds of genotypes BRRI dhan56, BRRI dhan48, and BRRI dhan71 were collected from Bangladesh Rice Research Institute (BRRI). Healthy and good-quality mature seeds were selected as explants and for treatment. Characteristics of rice varieties are as follows: BRRI dhan56 is medium salt tolerant and drought tolerant; BRRI dhan48 and BRRI dhan71 are both drought tolerant.

The seeds were dehusked, which were meant for germination. The dehusked seeds were soaked in 70% (v/v) ethanol and shaken for 5 min, followed by several washes. Then, the seeds were treated with 2% sodium hypochlorite (NaOCl) for 2-3 min followed by several washes. After that, they were treated with 0.1% (w/v) HgCl_2_ for 2-3 min with mild shaking and again washed with autoclaved distilled water several times. Finally, 2 drops of Tween 20 along with autoclaved distilled water were added. After mild shaking for 2 min, the solution was discarded and the seeds were subsequently washed with autoclaved distilled water.

Murashige and Skoog (MS) media was modified to make K^+^ sufficient medium and low K^+^ medium such as 1.5 mM MgSO_4_ was unaltered, 1.25 mM KH_2_PO_4_ and 2.99 mM CaCl_2_ were supplanted by 1.25 mM NH_4_H_2_PO_4_ and 2.99 mM Ca(NO_3_)_2_, and 20.6 mM NH_4_NO_3_ and 18.79 mM KNO_3_ were withdrawn. The final K^+^ concentration in the K^+^ sufficient medium and low-K^+^ medium were adjusted using KCl to 5 mM and 100 mM, respectively. On the other hand, media were modified from K^+^ sufficient medium for NO_3_^−^ treatment such as 1.5 mM MgSO_4_ and 1.25 mM NH_4_H_2_PO_4_ were unaltered, 2.99 mM Ca(NO_3_)_2_ was supplanted by 2.99 mM CaCl_2_ and afterward either 5 mM KNO_3_ or 5 mM KCl was included, respectively, representing NO_3_^−^ sufficient medium or NO_3_^−^ deficient medium. All the media contained 0.7% (w/v) agar and 3% (w/v) sucrose. Seeds and plantlets were maintained on sufficient or deficient medium at 25 °C under 16-h-light/8-h-dark photoperiod.

Dehusked seeds of all three varieties were grown on K^+^ and NO_3_^−^ sufficient media for 21 days. Then, they were transferred to low K^+^ (LK) and NO_3_^−^ deficient (LN) media and kept there for 2 days. Finally, plantlets were transferred back to K^+^ and NO_3_^−^ sufficient media. Data was taken during the whole process.

### Transcription analyses

For both RT-PCR and RT-qPCR analyses, total RNA was extracted from roots (150 mg) by using the TriZOL reagent (Invitrogen) and then treated with DNase I (Invitrogen™ DNA-free™ DNA Removal Kit) to eliminate genomic DNA contamination. The complementary DNA (cDNA) was synthesized by GoScript™ Reverse Transcription System (Promega Corporation, Madison, USA). Both oligo (dT) primers and random hexamer primers were used for RT-PCR and RT-qPCR analyses.

The eukaryotic elongation factor 1 alpha (*eEF-1α*) gene was used as an internal standard for normalization of gene expression levels [22]. Specifically designed primers were used to amplify *MYB59* transcript (403 bp): RT-PCR was performed for 35 cycles, each at 94 °C for 30 s, 55.7 °C for 50 s, and 72 °C for 1 min. Three independent experiments were performed. The list of primers with respective Tm values is shown in Supplementary Table S2.

RT-qPCR was conducted using a Maxima SYBR Green qPCR Master Mix (2X) (Thermo Fisher Scientific, Waltham, MA, USA) on a StepOne™ Real-Time PCR System machine (Applied Biosystems). The amplification reactions were performed in a total volume of 25 μl, which contained 12.5 μl SYBR Green Master Mix, 8.45 μl nuclease-free water, 0.75 μl forward and reverse primers (10 mM), and 2.5 μl cDNA. PCR was conducted as follows: 95 °C for 10 min, followed by 40 cycles of 95 °C for 15 s, 55.7 °C for 30 s, and 72 °C for 40 s. Three biological replicates were used in one independent RT-qPCR experiment. Three independent experiments were performed in one RT-qPCR analysis.

A melting curve analysis was performed at the end of the PCR run over a range of 65-95 °C. The ∆CT method using a reference gene was used for evaluating the gene expression levels. Finally, Student’s *t* test and graphs were plotted using GraphPad Prism 6.0.

## Results

### Conserved motif analysis

Using the MEME tool, we looked for the three most conserved motifs in 56 MYB59 proteins. Motif 1 was the longest, with 50 amino acid residues, followed by motif 2 with 24 and motif 3 with 29 residues (Supplementary Table S3). Long conserved residues present in the aligned sequences can demonstrate the conserved structures of MYB59 proteins among species.

### Multiple sequence alignment

Clustal Omega was used to align the MYB59 protein sequences from 56 plant species using the default parameters, where the residues were shaded as identical and similar. As mentioned earlier, we found 3 most conserved motifs with statistical significance. We highlighted only motif 1 and motif 2 in Supplementary Fig. S1 due to higher significance than motif 3. Motif 2 (LNRTGKSCRLRWVNYLHPGLKRGK) and motif 1 (MTPQEERLVLELHAKWGNRWSRIARKLPGRTDNEIKNYWRTHMRKKAQEK) from *Oryza sativa* indica were very similar to other plants and suggested that these conserved residues could potentially play a key role in the functionality of MYB59 and could be included in metabolic processes.

### Phylogenetic analysis of MYB59 proteins

**Fig. 1:**
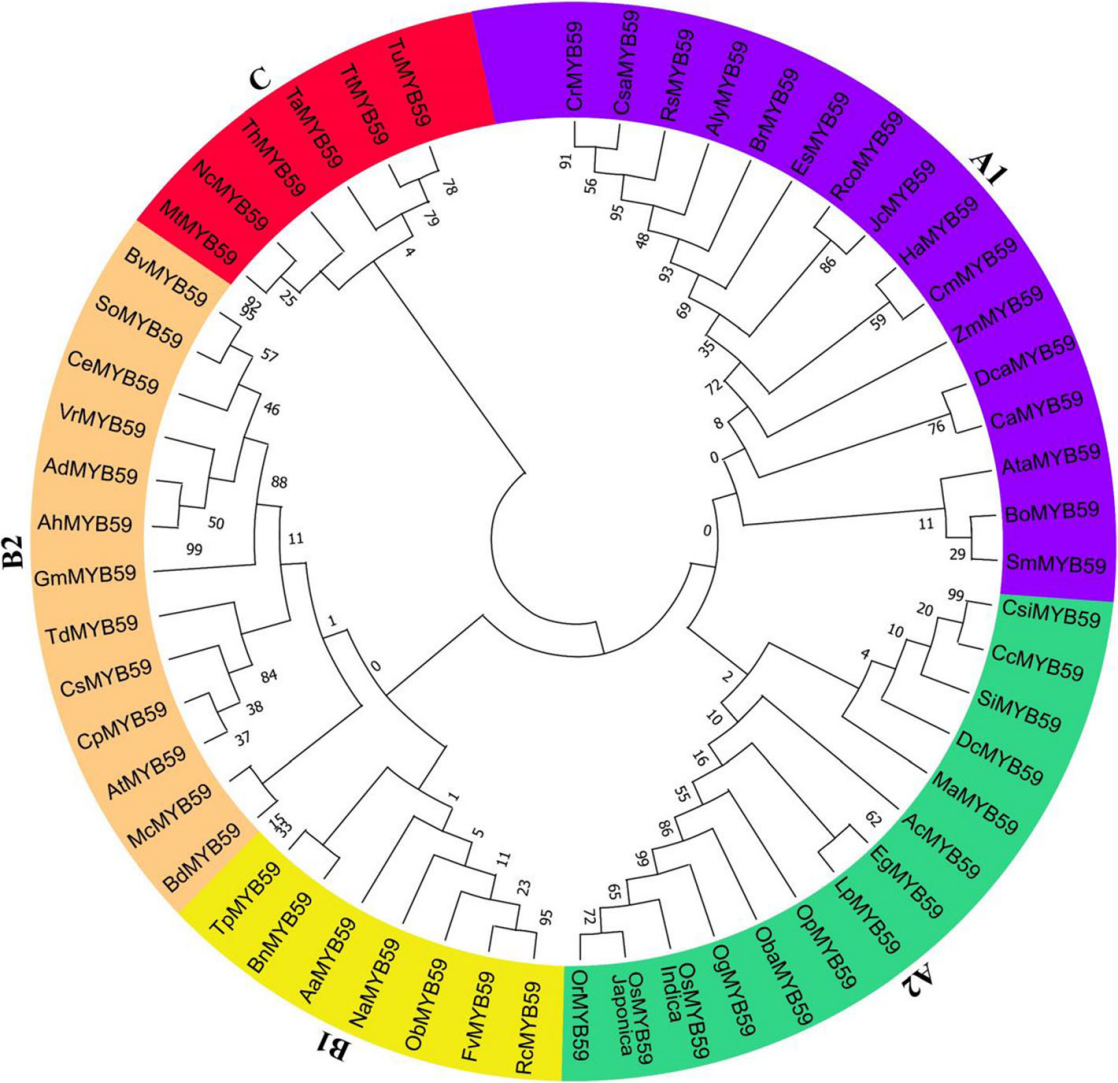
Phylogenetic relationships among MYB59 proteins from 56 plant species with bootstrap values

To comprehend the phylogenetic relationship among MYB59s, a maximum likelihood phylogenetic tree was developed, which incorporates both dicotyledonous plants and monocotyledonous plants. The constructed tree was divided into three primary groups based on the tree topology, for example, group A, B, and C. Based on the sub-clustering, group A was further subdivided into two subgroups such as A1 and A2. Group B was likewise subdivided into two subgroups on the basis of sub-clustering (B1 and B2) (Fig. 1). We found 6 homologs of genus *Oryza*, whereas MYB59s from different groups may differ from one another in terms of gene structure and conserved functional domains. However, even more molecular, genetic, and physiological studies on the functional role/s of MYB59 proteins under various perturbations are needed.

### Gene location and structure of *OsMYB59* indica

In this study, the genomic distribution of the *OsMYB59* indica in rice plants was investigated through chromosomal mapping. The gene was found on chromosome 1: 47, 136, 245-47, 137, 286 forward strand, as shown by the findings (Supplementary Fig. S2).

To establish the numbers and positions of exons and introns in the *OsMYB59* indica gene, an analogy of full-length cDNA sequences with the relevant genomic DNA sequences was made. The coding sequences of the *OsMYB59* indica gene were found to be interrupted by an intron (Supplementary Fig. S3).

### Cis-element analysis of rice *MYB59*

5′ UTR sequence (2000bp) of *OsMYB59* indica was subjected to classify possible regulatory cis-acting components in PlantCARE database. We found several cis-regulatory elements responsive to abiotic, biotic stresses, and light, as shown in Table 1.

**Table 1:** Predicted cis-elements in the promoter regions of the *OsMYB59* indica gene responsive to stresses and light

Cis-acting element	Sequence	Function	Source
ARE	AAACCA	Cis-acting regulatory element essential for the anaerobic induction	PlantCARE database
CGTCA-motif	CGTCA	Cis-acting regulatory element involved in the methyl jasmonate (MeJA) responsiveness	PlantCARE database
LTR	CCGAAA	Cis-acting element involved in low-temperature responsiveness	PlantCARE database
MYB	CAACAG	Stress-induced drought, low temperature, salt, abscisic acid, and gibberellic acid responses	(Zhu et al. 2005 [69])
MYC	CATGTG	Cold stress mitigation	(Maruyama et al. 2012 [37])
G-box	CACGAC	Cis-acting regulatory element involved in light responsiveness	PlantCARE database
I-box	gGATAAGGTG	Part of a light responsive element	PlantCARE database
Sp1	GGGCGG	Light responsive element	PlantCARE database
TCCC-motif	TCTCCCT	Part of a light responsive element	PlantCARE database
TCT-motif	TCTTAC	Part of a light responsive element	PlantCARE database

### Analysis of protein features

OsMYB59 indica has 276 amino acid residues with a molecular weight of 31.05 kDa and displayed primarily basic characteristics with 6.86 pI value. The subcellular localization of OsMYB59 indica was predicted as nuclear.

### 3D structure and DNA-binding prediction of OsMYB59 indica

**Fig. 2:**
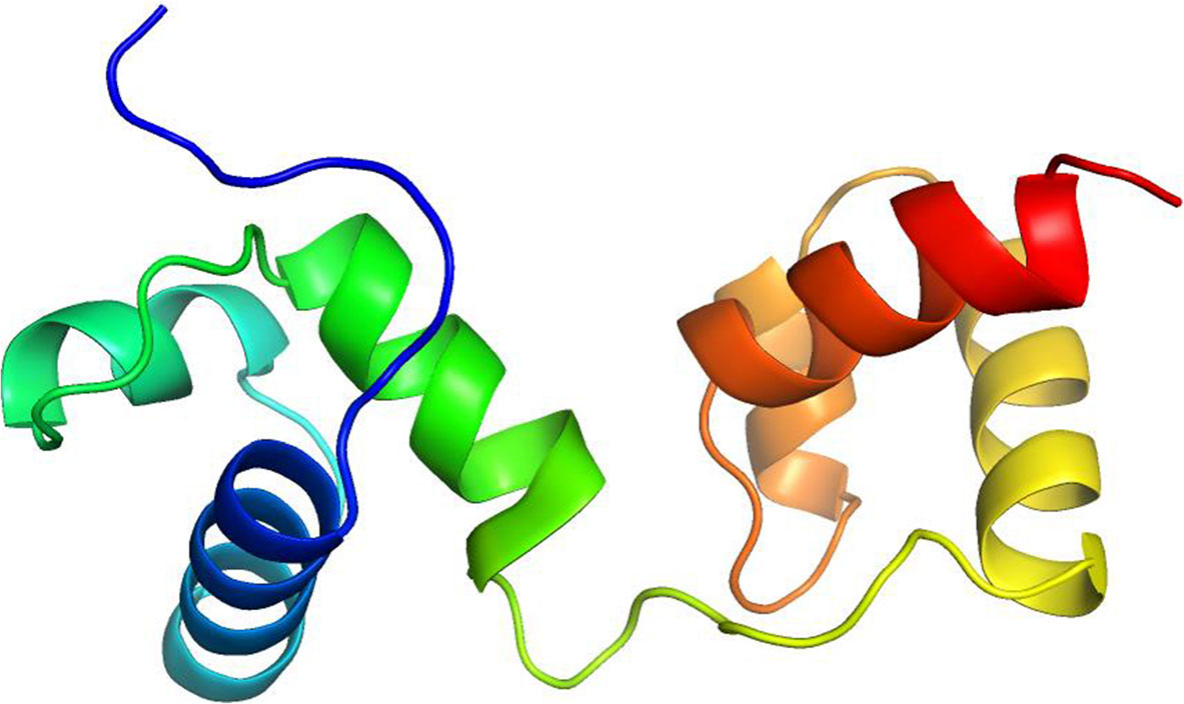
3D model of OsMYB59 indica protein. Models were generated by SWISS-MODEL, refined by ModRefiner, validated by Ramachandran plot, and visualized by PyMol

Three-dimensional models were generated by SWISS-MODEL; the model was refined using ModRefiner, and the quality of the model was analyzed using the Ramachandran plot, in which our model showed 98.9% in allowed residues and ERRAT quality factor was A: 100. *OsMYB59* indica gene synthesizes a DNA binding R2R3 (Repeat2 Repeat3) transcription factor, containing R2 and R3 amino acid sequence repeats (Fig. 2).

**Fig. 3:**
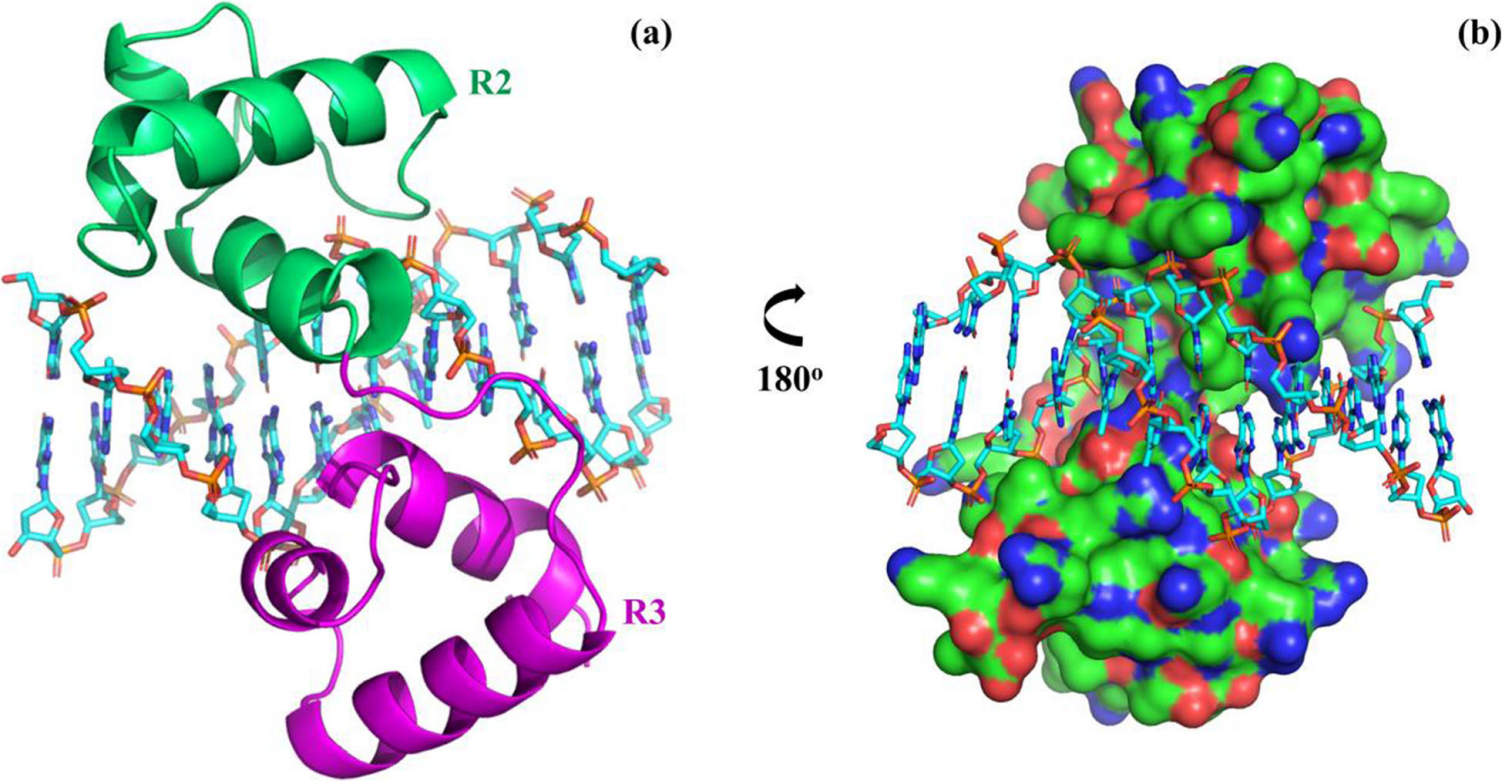
Predicted structure of the OsMYB59–DNA complex. DNAs are shown as sticks and OsMYB59 is shown as cartoon (**a**) and as an electrostatic surface potential map (**b**)

**Fig. 4:**
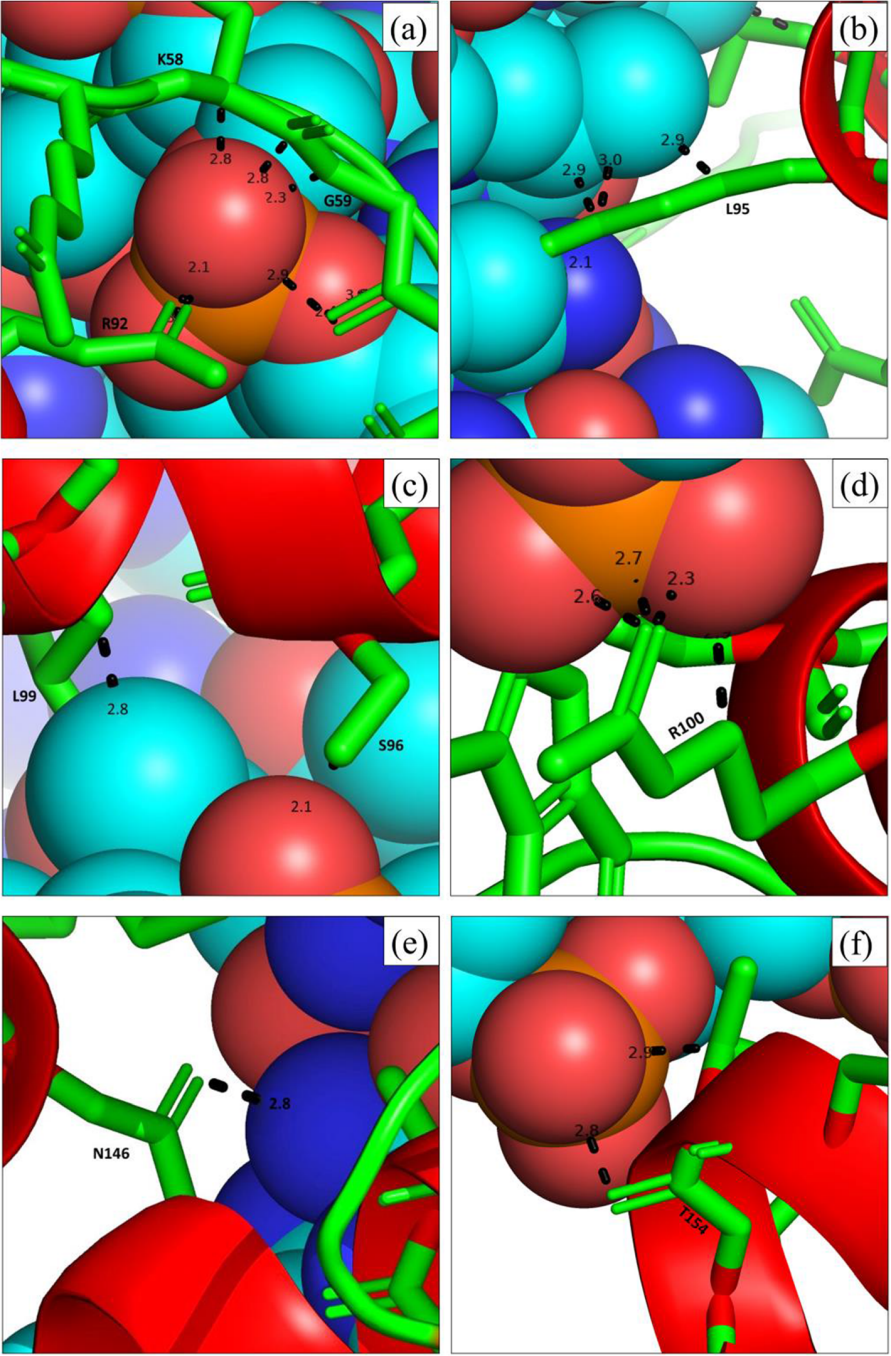
Predicted binding-site residues on protein OsMYB59 within 3Å. OsMYB59 is shown as cartoon-and-stick form and the DNA is shown as spheres. Here, (**a**) shows K58, G59, and R92, (**b**) shows L95, (**c**) shows S96 and L99, (**d**) shows R100, (**e**) shows N146, and (**f**) shows T154 residue

MYB59 protein and DNA (B-DNA) docking was done using HDOCK SERVER, where the docking score was −217.99 (Fig. 3). After docking, the binding-site residues on the protein (within 3Å) were visualized using PyMOL. We found 9 binding-site residues within 3Å and they were K58, G59, R92, L95, S96, L99, R100, N146, and T154 (Fig. 4).

### Morphological changes in treatment plants

**Fig. 5:**
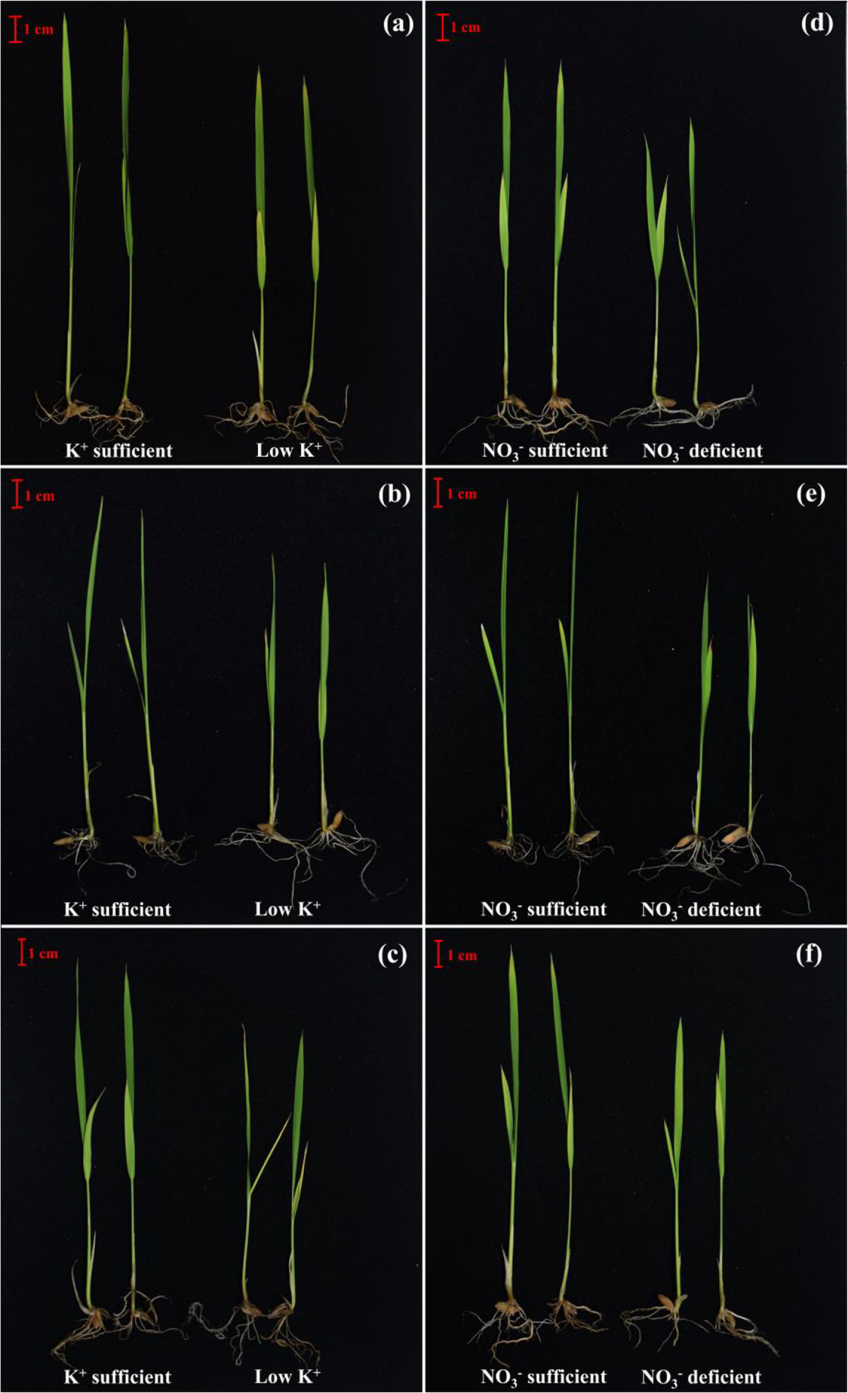
Morphology of the plants grown into K^+^ sufficient/low K^+^ medium in BRRI dhan56 (**a**), BRRI dhan48 (**b**), BRRI dhan71 (**c**), and NO_3_^−^ sufficient/NO_3_^−^ deficient medium in BRRI dhan56 (**d**), BRRI dhan48 (**e**), BRRI dhan71 (**f**); bar = 1cm

Plants were germinated on petridish and transferred to K^+^/NO_3_^−^ sufficient media or low K^+^ (LK)/NO_3_^−^ deficient (LN) media for monitoring the morphological changes. They were transferred after reaching two leaves stage and treated for 7 days. We found yellowish shoots when grown on LK and LN medium. In LK medium, a series of leaf color changes occurred, depending on variety, including yellow and a tan/brown color resulting from leaf death. In severe cases, leaf roll back and die-back (from leaf tips) will proceed. Older leaves or whole plants became yellowish-green in LN medium. Furthermore, older leaves and occasionally all leaves were light green and chlorotic at the tip apart from new greener leaves. The yellowing suggests potassium and nitrate deficiency causing chlorosis, reduced chlorophyll content, and slow growth (Fig. 5).

### RT-PCR analysis of the target and housekeeping gene

RT-PCR analysis showed the presence of both *OsMYB59* indica and *eEF-1α* bands. We found size specific bands in all three rice varieties and both treatments. Product sizes of *MYB59* and *eEF-1α* were 403 bp and 103 bp, respectively.

### Quantitative reverse transcription PCR (RT-qPCR) analysis of *OsMYB59* indica gene in responses to various treatments

Transcription factor MYB59 expression levels were evaluated using RT-qPCR after low K^+^ or NO_3_^−^ treatment. Plants germinated on K^+^/NO_3_^−^ sufficient (5 mM) medium were used as controls. For treatment, they were then relocated to either low K^+^ (100 mM—LK) or low NO_3_^−^ (0 mM—LN) medium for 2 days. The plants were then returned to a medium with sufficient K^+^ or NO_3_^−^ (5 mM). For the RT-qPCR assay, roots were acquired at different times, as indicated. The assay was repeated for three different rice varieties with different levels of tolerance to abiotic stresses. Melt curve and amplification plots from the StepOne^TM^ software were used to verify the amplification and specificity of the amplicons.

**Fig. 6:**
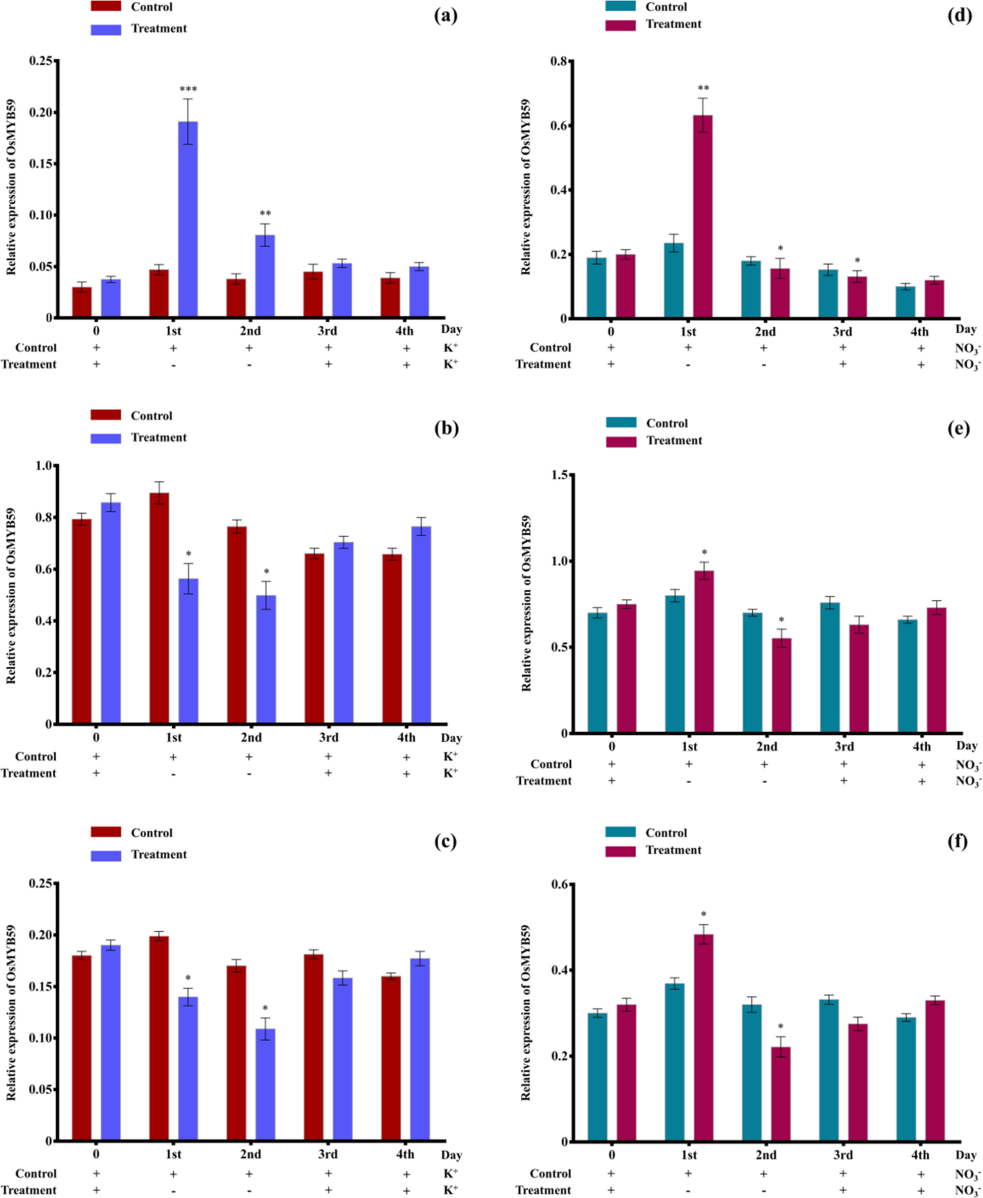
*OsMYB59* indica transcription responds to external K^+^ levels in BRRI dhan56 (**a**), BRRI dhan48 (**b**), BRRI dhan71 (**c**), and external NO_3_^−^ levels in BRRI dhan56 (**d**), BRRI dhan48 (**e**), and BRRI dhan71 (**f**). Plus (+) and minus (−) symbols represent sufficient and deficient conditions, respectively. RT-qPCR data are shown as means ± SE. Differences between the values of treatments and controls were compared using *t* tests (*p < 0.05, **p < 0.01, and ***p < 0.001)

BRRI dhan56 is a moderate salt and drought-tolerant variety [1, 24]. We saw upregulation of *OsMYB59* in both LK and LN medium for this variety. There is a significant increase in the *OsMYB59* transcript level on the 1st day of treatment which decreases rapidly in the subsequent days of treatment and the return back to sufficient medium (Fig. 6). However, we found different scenarios in the case of BRRI dhan48 and BRRI dhan71. These two varieties are drought tolerant and developed for Barind Tract of Bangladesh. For both BRRI dhan48 and BRRI dhan71, there is a slight decrease in the *OsMYB59* transcript level on the 1st and 2nd day of low K^+^ treatment, which then increases when returned back to sufficient medium. On the other hand, for low NO_3_^−^ treatment, *OsMYB59* transcript level follows, which was seen in the case of BRRI dhan56 (Fig. 6). With the increasing reaction temperature, double-stranded DNA, with dye molecules, is divided into single-stranded DNA that allows fluorescence to shift and a melting curve to form. In our study, the melt curves prove that only *MYB59* and *eEF-1α* were amplified with the melting temperature of ~87 °C for *MYB59* and ~79.5 °C for *eEF-1α*.

## Discussion

In order to assess the evolutions within the rice and other species MYB59 proteins, we have performed a phylogenetic study. There is a comparatively long evolutionary distance between *Arabidopsis* and rice, but we found close distance among the members of the genus *Oryza.* Consequently, its members’ developmental ties are more conservative than those of other subgroups.

The conserved functional domains we found from MEME analysis can have fundamental roles in different groups of MYB59 proteins. The gene structure, however, varied between different plant species. Therefore, they may have separate downstream regulatory genes and take part in various signaling paths. We found that *Arabidopsis* and japonica rice has no introns in their gene structure, but indica rice has one intron.

*OsMYB59* indica functions may be specified by the arrangements and nature of cis elements on the gene promoter. *OsMYB59* indica is primarily active in biotic, abiotic stresses, and light-inducing responsiveness, as seen by the findings. To date, only a few researches on functional analysis of the plant transcription factor OsMYB59 have been carried out. Light causes the plant transcriptome to be massively reprogrammed, increases or decreases gene expression and thus regulates the associated signaling pathway. MYB59 in *Arabidopsis* is classified as an R2R3 transcription factor. An earlier report found that MYB59 contributes to the regulation of cell cycle progression and root formation [38]. Prior studies showed a high resemblance in gene structure and amino-acid sequence between MYB59 and its nearest homolog MYB48 [63]. Furthermore, both play a role in the jasmonic acid signaling network, which indicates their operational redundancy [19]. Rice MYB59 also applies to the R2R3 type MYB protein and includes cis-regulatory elements that are sensitive to methyl jasmonate (MeJA) [46]. This indicates that both *Arabidopsis* and rice may have common functions for MYB59.

Although the majority of the residues associated with DNA binding are conserved, one residue (Leu versus Glu) of the R2 repeat significantly contrast among plant and animal MYB proteins, which likely contributes to the variation in their target DNAs. 5mC, as well as 6mA modifications, prohibit the interactions between MYB transcription factors and their target DNAs. The key residues responsible for DNA recognition are profoundly preserved in all R2R3-MYB proteins [53]. In addition, MYB transcription factors and DNA 5mC modifications show opposite functions in many plant specific processes, particularly fruit maturing [26, 33]. The buildup of anthocyanins in apple peel is negatively associated with DNA methylation levels, but contrarily it is positively related to MYB proteins [13]. Numerous R2R3-MYB proteins have been shown to increase anthocyanin [5, 6, 30, 41, 68], however, DNA hypermethylation brought about colorless or non-ripening fruits [35]. Significantly, the L→A mutation in WER (a MYB protein) resulted in stronger binding affinity than that of wild-type WER [53]. In addition, the DNA 6mA modification level is higher in plants compared to mammals. In rice, around 0.2% of adenines are modified by 6mA [66] and a comparative level was also seen in *Arabidopsis* [31]. DNA 5mC and 6mA modifications may direct gene expression by debilitating the interaction between MYB transcription factors and their target DNAs during plant development [53].

In plant’s responses to the K^+^ deficiency, transcriptional control is a very essential method. Under K^+^ deficient conditions, for example, the transcript levels of K^+^ transporter genes are upregulated to improve high affinity K^+^ intake of the plant roots [54]. Simultaneously, K^+^ transport from root to shoot should be decreased to retain enough K^+^ in the root. This represents an effective adaptive mechanism for maintaining root operation and the root to shoot K^+^ equilibrium. In order to preserve the required membrane potential and osmotic balance, the root must maintain a minimum concentration of K^+^ in the event of K^+^ deficiency in the soil. In comparison, the stem and young leaves reflect a large sink that needs a significant amount of K^+^ if adequate K^+^ is provided for plants’ development. As such, more K^+^ is carried to the shoots to promote plant growth. The root-to-shoot transportation of K^+^ then must be fine-tuned to external K^+^ levels. The yield and quality of crops depend on the sufficient supply of potassium and nitrogen. In certain agricultural regions like Asia, however, existing fertilization activity limits agricultural production and leads to several challenges.

In addition to a substantial reduction in nitrogen utilization efficiencies, the excessive usage of nitrogen fertilizer and the absence of potash fertilizers often lead to air/water/soil pollution and deterioration of the environment [18, 43, 65]. The coordination of the K/N use is the molecular mechanism behind this phenomenon. A previous study of *Arabidopsis* MYB59 showed that under sufficient K^+^/NO_3_^−^, MYB59 associates directly with the *NPF7.3* promoter and enables root to shoot K^+^/NO_3_^−^ transportation via NPF7.3. If plants suffer from a K^+^/NO_3_^−^ deficiency, *MYB59* will be downregulated and the *NPF7.3* transcript level will be reduced. The root to shoot transport of K^+^/NO_3_^−^ is therefore limited [11]. In our study, we found that under low K^+^ stress, two out of three selected varieties show downregulation of *MYB59* transcripts. These two varieties (BRRI dhan48 and BRRI dhan71) are drought tolerant. On the other hand, BRRI dhan56, which is moderate salt and drought tolerant, showed upregulation of *MYB59*. This suggests *MYB59* transcript level under low K^+^ also depends on the genotype and level of stress tolerance of the variety. Again, under low NO_3_^−^ stress, all three varieties showed upregulation of *MYB59*. Therefore, MYB59 possibly regulate one or more of K^+^ transporters like OsHAK, OsKAT, OsHKT [20, 40, 42, 51, 61], and NO_3_^−^ transporters like OsNPF2.4, OsNPF2.2, OsNAR2.1, and OsNRT2.3a [14, 34, 56, 59] in rice under stresses. In summary, our findings have helped to clarify the biological functions of rice MYB59 indica and established a theoretical framework to characterize its biological functions further in exposure to abiotic stress.

## Conclusions

As the world population increases, rice consumption has placed agriculture on top of the international agenda. To meet the dual challenge of producing enough food and alleviating poverty, more rice needs to be produced at a low unit cost so that the environment and ecosystem services can be safeguarded. At the same time, increased water scarcity in irrigated systems, as well as droughts, salinity, submergence, and global warming, are putting the capacity of rice-producing productive environments in jeopardy.

In our study, we characterized transcription factor MYB59 and analyzed its expression under low K^+^ and NO_3_^−^ stress. Transcription factor MYB59 participates in many plant biological processes but has not been systematically studied in rice. We found many cis-regulatory elements that confirm its participation under biotic and abiotic stresses. MYB59s from different groups diverged in terms of gene structure and conserved domains. The prediction of conserved motifs and domains, chromosomal and subcellular localization, and their sequence homology with others provided insight into the structure and putative functions. Real-time quantitative PCR analysis of the *OsMYB59* gene subjected to various stressors revealed that they are induced in response to external K^+^ and NO_3_^−^ levels and depend on the stress responsiveness of the drought and salt-tolerant varieties. During K^+^ stress, we found that *OsMYB59* is upregulated in BRRI dhan56 (about 3.1-fold increase vs control at 1st day of treatment), but downregulated in BRRI dhan48 (0.37-fold decrease vs control at 1st day of treatment), and BRRI dhan71 (0.3-fold decrease vs control at 1st day of treatment). On the other hand, *OsMYB59* is upregulated under NO_3_^−^ stress in all the three varieties (1.72-fold, 0.19-fold, and 0.33-fold increase vs control at 1st day of treatment in BRRI dhan56, BRRI dhan48, and BRRI dhan71, respectively). Previous studies showed only downregulation of OsMYB and AtMYB59 transcription factors under low K^+^, and upregulation of AtMYB59 under NO_3_^−^ deficient conditions. However, these responses are also regulated by light and individual *OsMYB59* expressions differed with the presence or absence of light. The availability of this information might encourage researchers for further functional validation.

## Supplementary Information

**Additional file 1: Supplementary Table S1.** List of retrieved MYB59 protein sequences from 56 plants species using NCBI and EnsemblPlants. **Supplementary Table S2.** List of primers with Tm and product size used in this study. **Supplementary Table S3.** Most conserved three motifs of MYB59 proteins in 56 plant species detected by using MEME tool. **Supplementary Fig. S1.** Multiple sequence alignments of the MYB59 proteinsobtained with Clustal Omega. Identical and similar residues were shaded as black and grey, respectively. Shading of the multiple-alignment file was done using BoxShade by ExPASy. **Supplementary Fig. S2.** Location of rice *MYB59* gene on rice chromosome 1. **Supplementary Fig. S3.** Gene structure of rice *MYB59* indica. Exons, introns, and untranslated regions are marked by round red rectangles, black lines, and blue rectangles, respectively. The scale bar at the bottom estimates the lengths of the exons, introns, and untranslated regions.

## Data Availability

All data generated or analyzed during this study are included in this published article [and its supplementary information files].

## Abbreviations

BRRI: Bangladesh Rice Research Institute
SE: Standard error
bp: Base pair
PCR: Polymerase chain reaction
RT-PCR: Reverse transcriptase polymerase chain reaction
RT-qPCR: Quantitative reverse transcription polymerase chain reaction
MeJA: Methyl jasmonate
