# Supplementary material for: Characterization of transcription factor MYB59 and expression profiling in response to low K+ and NO3− in indica rice (Oryza sativa L.)
Source: J Genet Eng Biotechnol. 2021 Oct 26;19:167. doi: 10.1186/s43141-021-00248-6 (PMC8548439; doi:10.1186/s43141-021-00248-6)
Supplement: Supplementary file 1 — Additional file 1: Supplementary Table S1. List of retrieved MYB59 protein sequences from 56 plants species using NCBI and EnsemblPlants. Supplementary Table S2. List of primers with Tm and product size used in this study. Supplementary Table S3. Most conserved three motifs of MYB59 proteins in 56 plant species detected by using MEME tool. Supplementary Fig. S1. Multiple sequence alignments of the MYB59 proteinsobtained with Clustal Omega. Identical and similar residues were shaded as black and grey, respectively. Shading of the multiple-alignment file was done using BoxShade by ExPASy. Supplementary Fig. S2. Location of rice MYB59 gene on rice chromosome 1. Supplementary Fig. S3. Gene structure of rice MYB59 indica. Exons, introns, and untranslated regions are marked by round red rectangles, black lines, and blue rectangles, respectively. The scale bar at the bottom estimates the lengths of the exons, introns, and untranslated regions. [file 43141_2021_248_MOESM1_ESM.docx]

**Supplementary Table S1** List of retrieved protein sequence of MYB59 proteins of 56 plants species from NCBI and EnsemblPlants.

| **Name** | **Accession no** | **Organism** |
| --- | --- | --- |
| ZmMYB59 | NP_001149882 | *Zea mays* |
| OsMYB59_Japonica | XP_015621159 | *Oryza sativa* japonica |
| BdMYB59 | XP_003566622 | *Brachypodium distachyon* |
| SiMYB59 | XP_022680579 | *Setaria italica* |
| AcMYB59 | XP_020084973 | *Ananas comosus* |
| DcMYB59 | XP_020671735 | *Dendrobium catenatum* |
| AaMYB59 | JAT49613 | *Anthurium amnicola* |
| AhMYB59 | XP_025641905 | Arachis hypogaea |
| GmMYB59 | XP_025980194 | *Glycine max* |
| CsiMYB59 | XP_006488255 | *Citrus sinensis* |
| RcMYB59 | XP_024159255 | *Rosa chinensis* |
| SmMYB59 | AGN52193 | *Salvia miltiorrhiza* |
| CmMYB59 | AMQ09367 | *Chrysanthemum x morifolium* |
| CeMYB59 | XP_027182269 | *Coffea eugenioides* |
| RcoMYB59 | XP_002531120 | *Ricinus communis* |
| CcMYB59 | XP_006424748 | *Citrus clementina* |
| EsMYB59 | XP_006400918 | *Eutrema salsugineum* |
| CrMYB59 | XP_023633463 | *Capsella rubella* |
| McMYB59 | XP_022148320 | *Momordica charantia* |
| SoMYB59 | XP_021865245 | *Spinacia oleracea* |
| AdMYB59 | XP_015952297 | *Arachis duranensis* |
| AlyMYB59 | XP_020871232 | *Arabidopsis lyrata* |
| JcMYB59 | XP_012064973 | *Jatropha curcas* |
| CsaMYB59 | XP_010455510 | *Camelina sativa* |
| BvMYB59 | XP_010685834 | *Beta vulgaris* |
| RsMYB59 | XP_018472596 | *Raphanus sativus* |
| ThMYB59 | XP_010535528 | *Tarenaya hassleriana* |
| CaMYB59 | XP_016561472 | *Capsicum annuum* |
| EgMYB59 | XP_012832083 | *Erythranthe guttata* |
| FvMYB59 | XP_011461278 | *Fragaria vesca* |
| NcMYB59 | JAU20512 | *Noccaea caerulescens* |
| AtMYB59 | AT5G59780 | *Arabidopsis thaliana* |
| BnMYB59 | CDX81554 | *Brassica napus* |
| BoMYB59 | Bo7g113550 | *Brassica olerace* |
| BrMYB59 | Bra002533 | *Brassica rapa* |
| CsMYB59 | KGN65673 | *Cucumis sativus* |
| DcaMYB59 | KZM95640 | *Daucus carota* |
| HaMYB59 | OTG28055 | *Helianthus annuus* |
| MtMYB59 | AES67579 | *Medicago truncatula* |
| MaMYB59 | GSMUA_Achr6T15370_001 | *Musa acuminata* |
| NaMYB59 | OIT27282 | *Nicotiana attenuata* |
| ObaMYB59 | OBART01G45730 | *Oryza barthii* |
| ObMYB59 | OB01G54920 | *Oryza brachyantha* |
| OgMYB59 | ORGLA01G0394700 | *Oryza glaberrima* |
| OsMYB59_Indica | BGIOSGA005283-TA | *Oryza sativa* indica |
| OpMYB59 | OPUNC01G44650 | *Oryza punctata* |
| OrMYB59 | ORUFI01G49220 | *Oryza rufipogon* |
| TpMYB59 | Tp57577_TGAC_v2_mRNA35471 | *Trifolium pratense* |
| TaMYB59 | TraesCS3B02G612200 | *Triticum aestivum* |
| TdMYB59 | TRIDC3AG075700 | *Triticum dicoccoides* |
| TtMYB59 | TRITD3Bv1G281370 | *Triticum turgidum* |
| TuMYB59 | TRIUR3_22432-T1 | *Triticum urartu* |
| VrMYB59 | Vradi02g05100 | *Vigna radiata* |
| AtaMYB59 | AET3Gv21248100 | *Aegilops tauschii* |
| LpMYB59 | LPERR01G40120 | *Leersia perrieri* |

**Supplementary Table S2** List of primers with Tm and product size used in this study.

| **Primer** | **Sequence** | **Tm(^o^C)** | **Product Size** |
| --- | --- | --- | --- |
| *MYB59* | F: 5’-ATCGCCAAGAGCATTCCTG-3’ | 55.4 | 403bp |
|  | R: 5’-AATCCGAGCAGAAGAAGGC-3’ | 55.0 |  |
| *eEF-1α* | F: 5’-TTTCACTCTTGGTGTGAAGCAGAT-3’ | 56.7 | 103bp |
|  | R: 5’-GACTTCCTTCACGATTTCATCGTAA-3’ | 55.0 |  |

**Supplementary Table S3** Most conserved three motifs of MYB59 proteins in 56 plant species detected by using MEME tool.

| **Motif** | **Width** | **Sites** | **E value** | **Sequence** |
| --- | --- | --- | --- | --- |
| 1. | 50 | 54 | 9.5e-2239 | MTPQEERLVLELHAKWGNRWSRIARKLPGRTDNEIKNYWRTHMRKKAQEK |
| 2. | 24 | 52 | 7.9e-1136 | LNRTGKSCRLRWVNYLHPGLKRGK |
| 3. | 29 | 44 | 6.0e-987 | RKGPWTEQEDVQLVWFVRLFGDRRWDFJA |

**
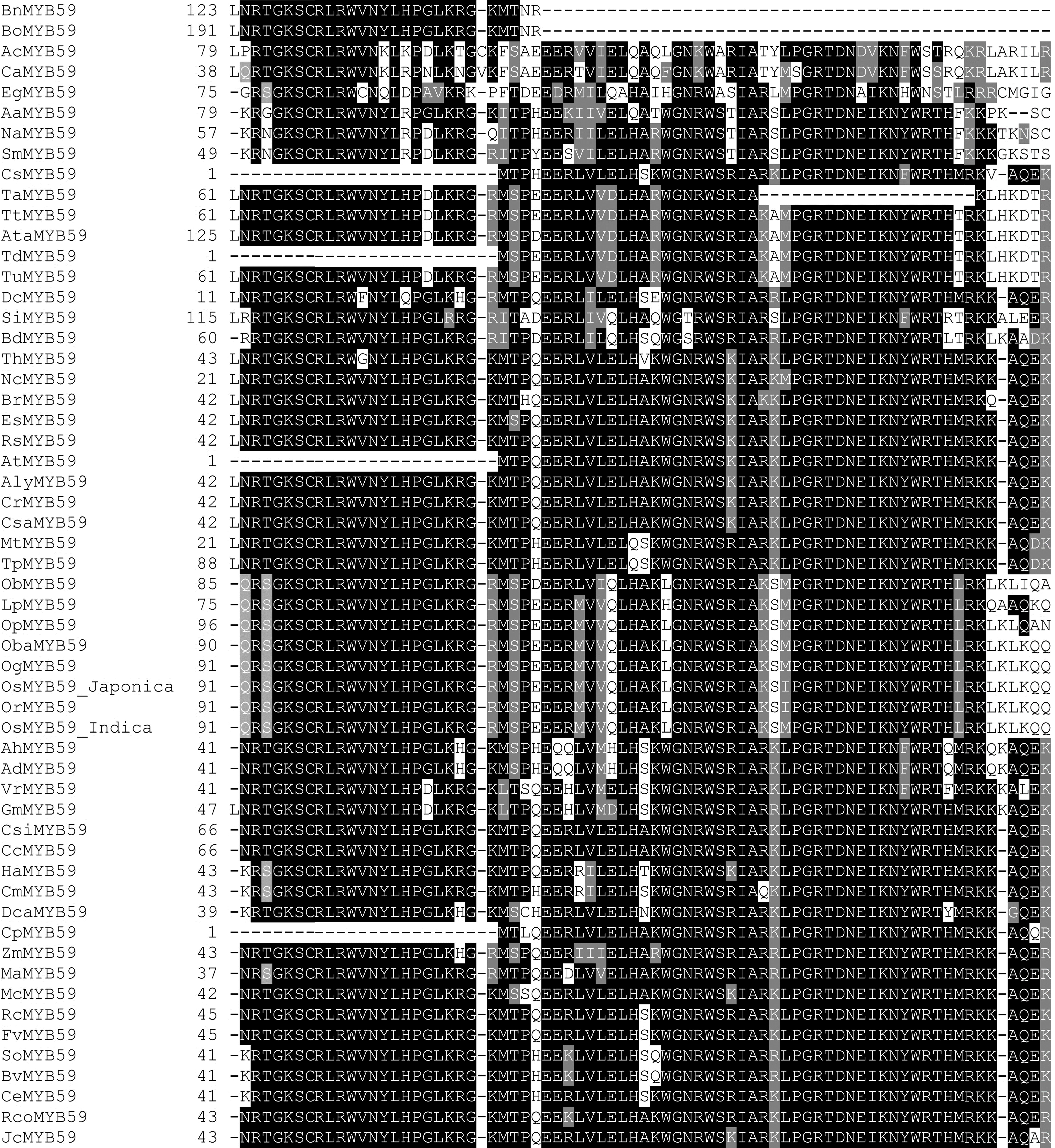
**

**Supplementary Fig. S1** Multiple sequence alignments of the MYB59 proteinsobtained with Clustal Omega. Identical and similar residues were shaded as black and grey, respectively. Shading of the multiple-alignment file was done using BoxShade by ExPASy.

**

**

**Supplementary Fig. S2** Location of rice *MYB59* gene on rice chromosome 1.

**
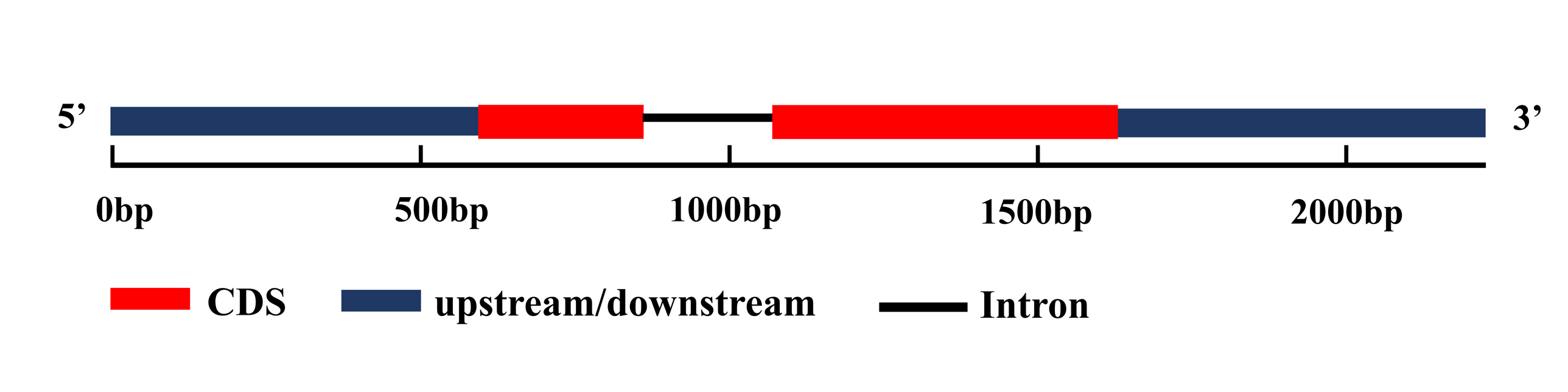
**

**Supplementary Fig. S3** Gene structure of rice *MYB59* indica. Exons, introns, and untranslated regions are marked by round red rectangles, black lines, and blue rectangles, respectively. The scale bar at the bottom estimates the lengths of the exons, introns, and untranslated regions.
